# Prevalence and Predictors of Health-Related Internet and Digital Device Use in a Sample of South Asian Adults in Edmonton, Alberta, Canada: Results From a 2014 Community-Based Survey

**DOI:** 10.2196/20671

**Published:** 2021-01-08

**Authors:** Mark J Makowsky, Charlotte A Jones, Shahnaz Davachi

**Affiliations:** 1 Faculty of Pharmacy and Pharmaceutical Sciences University of Alberta Edmonton, AB Canada; 2 Faculty of Medicine Southern Medical Program University of British Columbia Okanagan Campus Kelowna, BC Canada; 3 Primary Health Care Alberta Health Services Calgary, AB Canada

**Keywords:** consumer health information, cardiovascular disease, type 2 diabetes, eHealth, mobile phone, ethnicity, cross-sectional survey, Canada

## Abstract

**Background:**

South Asian Canadians are at high risk of developing cardiovascular disease and diabetes. Consumer-oriented health information technology may help mitigate lifestyle risk factors and improve chronic disease self-management.

**Objective:**

This study aims to explore the prevalence, patterns, and predictors of the use of the internet, digital devices, and apps for health purposes as well as preferences for future use of eHealth support in South Asian Canadians.

**Methods:**

We conducted a cross-sectional, mixed-mode survey in a convenience sample of 831 South Asian adults recruited at faith-based gathering places, health care settings, and community events in Edmonton, Alberta, in 2014. The 706 responders (mean age 47.1, SD 17.6 years; n=356, 50.4% female; n=509, 72.1% Sikh) who provided complete sociodemographic information were included in the analysis, and the denominators varied based on the completeness of responses to each question. Multivariate logistic regression was used to determine sociodemographic and health status predictors of internet use, being a web-based health information seeker, smartphone or tablet ownership, health app use, and willingness to use various modes of eHealth support.

**Results:**

Of all respondents, 74.6% (527/706) were internet users and 47.8% (336/703) were web-based health information seekers. In addition, 74.9% (527/704) of respondents owned a smartphone or tablet and 30.7% (159/518) of these had a health and fitness app. Most internet users (441/527, 83.7%) expressed interest in using ≥1 mode of eHealth support. Older age, being female, having less than high school education, preferring written health information in languages other than English, and lacking confidence in completing medical forms predicted lack of internet use. Among internet users, factors that predicted web-based health information seeking were being female, use of the internet several times per day, being confident in completing medical forms, and preferring health information in English. Predictors of not owning a smartphone or tablet were being older, preferring health information in languages other than English, having less than high school education, living in Canada for <5 years, having a chronic health condition, and having diabetes. Increasing age was associated with lower odds of having a health app. Preferring health information in languages other than English consistently predicted lower interest in all modes of eHealth support.

**Conclusions:**

eHealth-based chronic disease prevention and management interventions are feasible for South Asian adults, but digital divides exist according to language preference, education, age, sex, confidence in completing medical forms, and number of years lived in Canada. Community-based, culturally tailored strategies targeting these factors are required to address existing divides and increase the uptake of credible web-based and app-based resources for health purposes.

## Introduction

South Asians originating from India, Pakistan, Bangladesh, and Sri Lanka are among the fastest growing and largest visible minority groups in Canada [1]. Cardiovascular disease (CVD) and diabetes are among the most prevalent health problems facing South Asians regardless of whether they live in their country of origin or abroad [2]. Recent reviews have highlighted that South Asian migrants in Canada have 1.5 to 2 times the prevalence of coronary artery disease compared with age- and sex-adjusted Whites of European ancestry [2-4]. New cases of CVD disproportionally affect younger South Asian individuals. This was demonstrated in a large, international case-control study where the median age of first myocardial infarction in South Asians (53 years) was 6 to 10 years younger than those in North America or Western Europe [5].

The increased risk of coronary artery disease is primarily driven by a higher incidence of known atherosclerotic CVD risk factors, particularly type 2 diabetes and impaired glucose tolerance [6]. Both biological and nonbiological mechanisms are implicated in the increased risk of coronary artery disease and diabetes. For example, a recent meta-analysis found that South Asian Canadians had a higher prevalence of type 2 diabetes, hypertension, abdominal obesity, percentage body fat, increased carbohydrate intake, and sedentary lifestyle [3]. Individual studies have shown that South Asians are 2 to 3 times more likely to develop type 2 diabetes compared with other populations and develop diabetes at a younger age; approximately 4.6 years younger than Chinese or White Canadians [7-10]. Differences in genetic factors may explain some of the increased rates of CVD risk factors, but existing evidence suggests that the biology of CVD is no different in South Asians compared with other ethnic groups [6]. Nonbiologic mechanisms, including acculturation, a shift from traditional dietary habits, physical inactivity, other environmental factors (eg, psychosocial stress, social support), and access to health services, have all been implicated in the increased risk of CVD, diabetes, and other CVD-related risk factors [6,11,12].

Clinical practice guidelines recommend lifestyle management focusing on diet and physical activity, pharmacologic therapy, and self-management education in the primary prevention and management of CVD and diabetes and their associated risk factors [6,13,14]. Despite these recommendations, evidence suggests that risk factors and diabetes control are suboptimal in South Asian individuals [15]. Canadian data suggest that 55% of South Asian patients are above-recommended blood glucose A_1c_ targets, 36% exceed blood pressure targets, and 58% exceed lipid level targets [15]. Language barriers, sociocultural factors, limited diabetes and CVD awareness, lack of access to culturally tailored diet counseling, misconceptions around diet, perceptions around physical activity, and lower compliance with pharmacotherapy may contribute to the increased risk [2,16-18].

There has been large growth in consumer-oriented health information technology, such as Web 2.0, and app-based interventions supporting healthy lifestyles and the management of chronic health conditions [19]. Emerging evidence suggests that mobile health (mHealth), internet, and social media–based interventions may improve the prevention and management of chronic health conditions [20], cardiovascular risk factors including unhealthy diet and physical inactivity [21,22], and diabetes [23-25]. Several successful culturally tailored programs targeting diabetes and cardiovascular risk have been developed in Canada, but accessing these programs can be challenging [26-30]. The use of credible consumer-oriented eHealth resources by the South Asian community in Canada could increase access to and efficiency in the delivery of culturally tailored chronic disease self-management programs, which may further assist in the prevention and management of CVD and type 2 diabetes and their common risk factors and complications in this high-risk population.

Large, nationally representative surveys suggest high levels of digital device ownership [31], uptake of the internet [32], and web-based health information seeking in North America [33,34]. However, digital divides in internet use for health information related to sociodemographic factors and ethnicity [35] exist in the United States. There is limited information on use patterns and predictors of web-based health information–seeking behaviors and use of digital devices for health purposes among English- and Punjabi-speaking South Asian Canadians. This information is important and could be used to justify and inform the development of tailored consumer-oriented eHealth interventions. Such interventions may help to overcome identified gaps in the knowledge and skills needed to effectively apply high-quality web-based and mobile phone–based resources for the prevention and management of chronic conditions. This information could also be used to inform and assist clinicians on how to optimally engage individuals with existing web-based health information resources.

The objective of this study is to describe prevalence, patterns, and predictors of internet use for health purposes, ownership of digital devices, use of health and fitness apps, and preferences for different eHealth-based support tools in a sample of English- or Punjabi-speaking South Asian adults recruited from Edmonton, Alberta. Specifically, we explore the extent to which these variables are influenced by sociodemographic, health status, and technology use factors, including age, gender, education, health literacy, language preferences, and the presence of chronic health conditions.

## Methods

### Study Design

We used a community-based approach and worked in partnership with 13 faith-based, cultural, community, and health care organizations in a major metropolitan Canadian city, Edmonton, Alberta. We conducted a descriptive cross-sectional, mixed-mode anonymous survey. The survey was primarily delivered via a computer-assisted personal interview using the Qualtrics (Qualtrics Corporation) web-based survey platform. One-on-one interviews using paper-based surveys and an optional web-based version were also used.

### Participants, Recruitment, and Survey Administration

Participant recruitment occurred at 4 gurdwaras, 2 temples, 1 community pharmacy, 1 medical clinic, 2 community centers, and 2 large South Asian community events between May 18 and August 31, 2014. English- or Punjabi-speaking community members were eligible to participate if they were aged older than 18 years, self-identified their ethnic origins in India, Pakistan, Bangladesh, Nepal, or Sri Lanka, and were currently living in Alberta.

At community events, potential respondents were notified of the presence of the research team via announcements and posters. Potential respondents were then approached by community volunteers and presented with the survey information letter and asked if they would like to participate. If the potential participant agreed to participate, consent was implied and the survey was administered. Bilingual, trained community volunteers administered the survey in English or Punjabi according to respondents’ preference. Participants who felt comfortable using tablet computers self-administered the survey.

Potential participants who were unwilling to complete the in-person survey were invited to complete the survey on the web, which was also advertised using posters in the community, via social media and word of mouth. At selected survey locations, including the participating community pharmacy and family physician clinic, we attempted to recruit consecutive attendees. At these locations, the survey was conducted while waiting to have prescriptions filled or awaiting assessment. Respondents who completed the survey in person were offered a reusable shopping bag as an incentive and the opportunity to enter a draw for a tablet computer or various gift cards, whereas those who completed the survey on the web were only eligible to enter the draw.

### Survey Instrument

The e-Patient Project Survey evaluated the levels of digital device ownership, internet use, health information–seeking behaviors, health and fitness app use, levels of eHealth literacy, and preferences for participation in different modes of eHealth support (Multimedia Appendix 1). The research team developed the survey in 3 stages: literature review, key informant interviews with 16 individuals from the target communities, and a pilot test with 19 other individuals from the target communities. Most of the items were adopted from existing instruments, including the Pew Research Centre’s Internet & American Life Project 2012 Health survey [34,36,37], the 2012 Statistics Canada Canadian Internet Use Survey [33], the eHealth Literacy Scale [38], and a health literacy screening questionnaire [39].

The survey was translated into Punjabi according to the World Health Organization guidance for translation and adaptation of instruments [40]. One translator with a medical background who was fluent in both Punjabi and English conducted forward translation from English to Punjabi. Emphasis was placed on conceptual rather than literal translations. A panel of 2 bilingual community member reviewers further identified and reviewed inadequate expressions and concepts in the translated version. The back translation was conducted by a separate translator who was fluent in both English and Punjabi. Translation discrepancies were discussed and addressed by the project team.

### Measurement of Outcome Variables

We reported technology use outcomes as dichotomous variables. Individuals who answered affirmatively to either “Do you go online at least occasionally?” or “Do you send or receive email at least occasionally?” were characterized as internet users. Web-based health information seekers were those who indicated getting information about health on the internet or on the web. Individuals who answered affirmatively to “Is your cellphone a smartphone such as an iPhone, Android, Blackberry or Windows phone?” or “Do you own an iPad or other tablet computer such as an Android tablet, Microsoft surface or Kindle Fire?” were considered owners of a smartphone or tablet device. Owners of digital devices were asked about the use of health and fitness apps using one question, “On your smartphone or tablet, do you happen to have any health or fitness software apps (eg, track your food intake, weight, physical activity, or keep track of your medications).” We explored internet users’ preferences for the use of 6 different modes of eHealth support in the future, including (1) accessing a webpage includes a forum where you could connect with others like you, (2) accessing a YouTube channel for people with your condition(s) that has experts talking about best management, (3) using a smartphone app or wearable device that can monitor your condition, track your progress on your health goals, and/or provide reminders about when to take your medications, (4) following a specific Twitter account for your condition(s), (5) signing up for personalized text messages providing health updates or reminders for your condition(s), or (6) using a web-based education program. We adjusted the response options to those who indicated having at least one chronic condition, those who indicated they had diabetes, or those without a chronic condition.

### Measurement of Sociodemographic and Health Status Predictor Variables

Demographic factors included age, sex, education, marital status, duration of time lived in Canada, and the South Asian community with which respondents identified. Self-rated health status was assessed using a single question from the 36-item Short Form survey. The presence of 6 chronic health conditions was assessed by asking, “Have you ever been told by a doctor, nurse or other health care professional that you have, followed by the response options (eg, ‘diabetes or sugar disease’).” Language preference was assessed by asking, “In what language would you prefer to receive written health information?” and categories were collapsed into *includes English* or *does not include English*. One question “How confident are you filling out medical forms by yourself” estimated health literacy [39].

### Analysis

We limited the analysis to individuals who provided complete information on sociodemographic variables, language preference, health literacy, health status, and diabetes status variables. Surveys with missing data for other items were included in the analysis. Descriptive statistics were tabulated and depicted as the proportion of valid cases where incomplete responses for each outcome variable and *choose to not answer* or *don’t know* responses were considered missing. Descriptive data were analyzed using SPSS version 23 for Mac (IBM Corporation). Logistic regression was performed using R 3.1.3 (The R Foundation) to assess the effect of demographic and health factors on the dichotomous outcome variables. Variables shown to be statistically (*P*<.05) and clinically significant in the descriptive and univariate level analyses were selected to be included in the models. Self-rated health status was dropped for models that could not include all the factors. This was based on the widely used rule of thumb that there should be at least 10 events per parameter. This factor was dropped, as it was thought to be the least important. Other models included all variables. Multicollinearity was assessed by variance inflation factor (VIF), and VIF coefficients >10 were considered as high multicollinearity.

Goodness-of-fit, measuring the discrepancy between observed values and the expected value under the model, was assessed by using Craig and Uhler Pseudo R-square, Hosmer and Lemeshow goodness-of-fit test, and area under the curve (AUC). A *P* value <.05 indicated statistical significance. All the models fit reasonably well, as multicollinearity was not present in any model, all *P* values for the Hosmer-Lemeshow goodness-of-fit were >.05 (indicating no evidence of poor fit), and all AUC scores were greater than 0.7. However, most Craig and Uhler Pseudo R-square values were low (<0.5).

### Ethics Approval

The Health Research Ethics Board at the University of Alberta (Pro00038210) approved this study.

## Results

### Participant Flow

**Figure 1:**
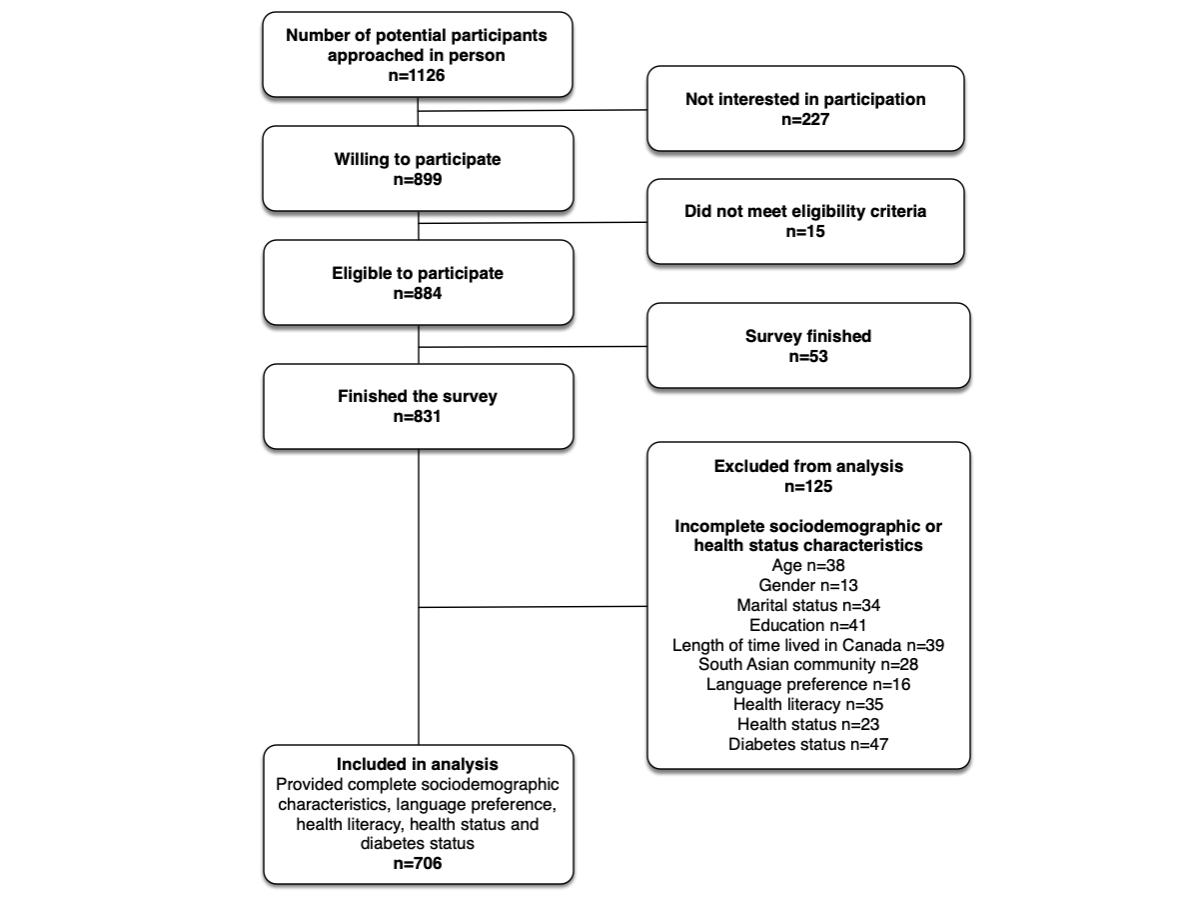
Flow diagram of the e-Patient Project Survey, Edmonton, Alberta, in 2014.

We approached 1126 potential participants for face-to-face surveys at community events and 831 agreed to complete the survey. A total of 706 individuals (706/831, 85.0%) provided complete sociodemographic and health status information (Figure 1).

### Participant Characteristics

The characteristics of the 706 study participants are shown in Table 1. Overall, the mean age was 47.1 (SD 17.6) years, and 50.4% (356/706) were female, 64.6% (456/706) had college or university education, and 72.1% (509/706) self-identified as Sikh. A total of 25.4% (179/706) of the participants lived in Canada for <5 years, and 31.0% (219/706) preferred written health information in a language other than English. Overall, 53.4% (377/706) of the participants self-reported at least one chronic health condition and 19.8% (140/706) reported diabetes. Most respondents rated their own health in the past 4 weeks as good (283/706, 40.1%) or very good (169/706, 23.9%), whereas 15.6% (110/706) and 3.7% (26/706) rated their health status as fair and poor, respectively. In addition, 11% (78/706) of the participants indicated being not at all confident in filling out medical forms on their own. Most respondents (397/706, 56.2%) were recruited at places of faith-based gathering and community events, whereas a minority (41/706, 5.8%) completed the survey on their own on the web.

**Table 1:** Demographic characteristics of the study sample.

Characteristics		Respondents	Missing data, n (%)
**Age (years;n=706)**			N/A^a^
	Mean (SD)	47.1 (17.6)	
	Median (IQR)	45 (32-63)	
**Age group (years; n=706), n (%)**			N/A
	18-34	213 (30.2)	
	35-49	181 (25.6)	
	50-64	156 (22.1)	
	≥65	156 (22.1)	
**Sex (n=706), n (%)**			N/A
	Male	350 (49.6)	
	Female	356 (50.4)	
**Marital status (n=706), n (%)**			N/A
	Not married	140 (19.8)	
	Married	566 (80.2)	
**Education (n=706), n (%)**			N/A
	Less than high school	63 (8.9)	
	High school	187 (26.3)	
	College, university, or higher	456 (64.6)	
**Lived in Canada (years; n=706), n (%)**			N/A
	>5	527 (72.1)	
	0-5	179 (25.4)	
**Community (n=706), n (%)**			N/A
	Sikh	509 (72.1)	
	Hindu	134 (19.0)	
	Other	63 (8.9)	
**Language preference (n=706), n (%)**			N/A
	English	487 (69.0)	
	Not English	219 (31.0)	
**Confidence in filling out medical forms (n=706), n (%)**			N/A
	Greater than not at all	628 (89.0)	
	Not at all	78 (11.0)	
**Health status (n=706), n (%)**			N/A
	Excellent	118 (16.7)	
	Very good	169 (23.9)	
	Good	283 (40.1)	
	Fair	110 (15.6)	
	Poor	26 (3.7)	
**Medical conditions, n (%)^b^**			
	Diabetes or sugar disease (n=706)	140 (19.8)	N/A
	High blood pressure (n=706)	178 (25.2)	N/A
	Heart disease (eg, angina, heart attack, or stroke; n=689)	48 (7.1)	17 (2.4)
	Lung conditions (eg, asthma or bronchitis; n=687)	41 (5.8)	19 (2.7)
	Arthritis (n=688)	124 (17.6)	18 (2.5)
	Cancer (n=684)	23 (3.4)	22 (3.1)
	Other chronic condition treated with daily medication (n=688)	110 (16.2)	18 (2.5)
	High cholesterol (n=608)^c^	144 (23.7)	98 (13.9)
**≥1 condition (n=706), n (%)**			N/A
	No	329 (46.6)	
	Yes	377 (53.4)	
**Location of recruitment (n=706), n (%)**			N/A
	Community setting	397 (56.2)	
	Health setting	268 (38.0)	
	On the web	41 (5.8)	

^a^N/A: not applicable.

^b^Data are n (%) out of 706 respondents unless there were missing data, in which case the n (%) of valid cases is reported.

^c^High cholesterol was unintentionally omitted from the paper version of the survey administered at the first community event.

### Internet Use, Sources of Health Information, and Web-Based Health Information–Seeking Behavior in Internet Users

Overall, 74.6% (527/706) of respondents were classified as internet users, whereas 25.4% (179/706) were nonusers (Table 2). Respondents used a median of 3 (IQR 2-5) different sources of health information, most commonly their doctor or health care provider (656/704, 93.2%) and family (398/702, 56.7%). Overall, 47.8% (336/703) of all respondents, or 63.4% (332/524) of internet users, used the internet for health information (Table 2). When asked how important it is to find health information tailored to their specific needs as a person of a South Asian background, 73.8% (513/695) indicated it was *very* or *extremely* important.

Patterns of use among the 527 internet users are shown in Multimedia Appendix 2. Most internet users (373/517, 72%) were on the web several times per day and most watched videos on YouTube, used social media sites, or made video calls. The most commonly reported web-based health information–seeking tasks were looking for information on healthy lifestyles (354/524, 67.6%) and on a specific disease or medical condition (248/460, 53.9%) and symptoms they were experiencing (222/523, 42.4%).

Regarding the use of Web 2.0 for health, just less than half of internet users (240/523, 45.9%) watched a web-based video about health or medical issues, 42.4% (222/523) read about someone else’s experience about health or medical issues in a blog, newsgroup, or website, and 29.5% (153/518) reported going on the web to find others who might have similar health concerns**.** Although there were significant missing data because of a problem with the printed version of the survey, just more than one-fourth of respondents indicated that web-based health information they found or someone else found for them affected a treatment decision (94/343, 27.4%), whereas more participants responded that it had led them to ask their doctor new questions, go to see their doctor, or change the way they maintain their health (Multimedia Appendix 2).

**Table 2:** Internet user status, sources of health information, digital device ownership, and health and fitness apps.

Characteristics			Overall, n (%)		Missing data, n (%)
**Internet use (n=706)**					
	Internet user	527 (74.6)		N/A^a^	
	Noninternet user	179 (25.4)		N/A	
**Where do you get information about health questions that you have?**					
	Doctor or health care provider (n=704)^b^	656 (93.2)		2 (0.3)	
	Family (n=702)	398 (56.7)		4 (0.6)	
	Internet (n=703)	336 (47.8)		3 (0.4)	
	Print (n=704)	309 (43.9)		2 (0.3)	
	Friends (n=703)	285 (40.5)		3 (0.4)	
	TV or radio (n=705)	283 (40.1)		2 (0.3)	
	Others with the same condition (n=639)	66 (10.3)		67 (9.5)	
	Never looked (n=618)	14 (2.3)		88 (12.5)	
**How important is it for you to find health information tailored to your needs as someone of South Asian background? (n=695)**					
	Extremely or very important	513 (73.8)		11 (1.6)	
**Device ownership (Do you own…)**					
	A desktop or laptop computer at home connected to the internet? (n=701)	615 (87.7)		5 (0.7)	
	A cellphone, iPhone, Blackberry, or other device that is a cellphone? (n=706)	571 (80.9)		N/A	
	Is your cellphone a smartphone? (n=571)	443 (77.6)		N/A	
	An iPad or other tablet computer (n=704)	376 (53.4)		2 (0.3)	
	Smartphone or tablet (n=704)	527 (74.9)		2 (0.3)	
**Device use (Do you use your smartphone or tablet to…)**					
	Send or receive text messages (n=527)	432 (82.0)		N/A	
	On your smartphone or tablet, do you happen to have any health or fitness apps? (n=518)	159 (30.7)		9 (1.7)	
**What type of health and fitness apps are you currently using? (n=138)**					
	Tracking food, diet, or calorie intake	88 (63.8)		21 (13.2)	
	Monitoring weight	49 (35.5)		21 (13.2)	
	Physical activity tracking	65 (47.1)		21 (13.2)	
	Track runs that you take	20 (14.5)		21 (13.2)	
	Mobile pedometer	32 (23.2)		21 (13.2)	
	Research or diagnose medical conditions	9 (6.5)		21 (13.2)	
	Keep track of medications	9 (6.5)		21 (13.2)	
	Stress management	17 (12.3)		21 (13.2)	
	Communicate with your doctor or health care provider	10 (7.2)		21 (13.2)	
	Monitor sleep cycle	12 (8.7)		21 (13.2)	
	Record your blood pressure	13 (9.4)		21 (13.2)	
	Record your blood sugar or diabetes	8 (5.8)		21 (13.2)	
	Other	6 (4.3)		21 (13.2)	

**^a^**N/A: not applicable.

^b^Data are n (%) out of 706 respondents unless otherwise specified. When there were missing data, the n (%) of valid cases was reported.

### Digital Device Ownership and Health and Fitness Apps in Smartphone or Tablet Owners

Overall, 62.8% (443/705) of respondents owned a smartphone, 53.4% (376/704) owned a tablet computer, and 74.9% (527/704) owned either a smartphone or tablet (Table 2). Most smartphone or tablet owners (432/527, 82%) reported sending or receiving text messages. Just less than one-third of the smartphone or tablet owners surveyed (159/518, 30.7%) indicated that they had a health and fitness app on their mobile device (Table 2). The most commonly used apps included those designed to track food, diet, or calorie intake (88/138, 63.8%), track physical activity (65/138, 47.1%), and monitor weight (49/138, 35.5%).

### Preferences for Future eHealth Interventions in Internet Users

Most internet users (441/527, 83.7%) responded that they were likely or very likely to use at least one of the 6 presented eHealth tools to address a health issue in the next 12 months (Multimedia Appendix 2). Although there were some systematic issues with missing information regarding YouTube, Twitter, and a web-based education program, most respondents favored accessing a YouTube channel (330/425, 77.6%) followed by using a webpage with peer-to-peer support (353/500, 70.6%), using an app or a wearable device (316/493, 64.1%), or receiving personalized text messages (282/483, 58.4%).

### Barriers in Nonusers of the Internet

The 179 respondents who were not internet users reported several barriers, the most common being *lack of skills* (114/177, 64.4) and *no interest* (72/177, 40.7%; Table 3)**.** One-third of nonusers (57/178, 32%) said they were planning to get on the web in the future. Of these, 67.3% (35/52) indicated being *likely* or *very likely* to attend a hands-on workshop, whereas 72.2% (38/52) were *likely* or *very likely* to learn from a friend or family member. Most internet nonusers (136/175, 77.7%) reported knowing someone who could help them get on the web.

**Table 3:** Barriers to nonusers of the internet.

Characteristics		Noninternet users, n (%)	Missing data, n (%)
**What are the reasons you do not go on web?^a^**			
	Lack of skills (n=177)	114 (64.4)	2 (1.1)
	No interest (n=177)	72 (40.7)	2 (1.1)
	Too late to learn (n=176)	38 (21.1)	3 (1.7)
	Limited access to a computer (n=176)	9 (5.1)	3 (1.7)
	Uncomfortable using a computer (n=175)	7 (4.0)	4 (2.2)
	Privacy reasons (n=176)	7 (4.0)	3 (1.7)
	Fear of technology (n=175)	5 (2.9)	4 (2.2)
	Because of disability (n=176)	4 (2.3)	3 (1.7)
	Cost (n=178)	3 (1.7)	1 (0.6)
**Are you likely to start going on web in the future?**			
	Yes, within 6 months (n=178)	23 (12.9)	1 (0.6)
	Yes, within 6 to 12 months (n=179)	16 (9.0)	N/A^b^
	Yes, in more than a year (n=179)	18 (10.1)	N/A
	Not likely (n=179)	61 (34.3)	N/A
	Never (n=179)	60 (33.7)	N/A
**Likeliness to use the following strategies to improve their ability to go on the web (n=57; who answered yes)**			
	Likely or very likely to attend a hands-on workshop (n=52)	35 (67.3)	5 (8.8)
	Likely or very likely to talk with a friend or family member (n=52)	38 (72.2)	5 (8.8)
Know someone who could help them, if they needed to go on web to do something (n=175)		136 (77.7)	4 (2.2)

^a^Data are n (%) out of 179 noninternet users unless otherwise specified. When there were missing data, the n (%) of valid cases was reported.

^b^N/A: not applicable.

### Predictors of Internet Use and Web-Based Health Information Seeking

As shown in Multimedia Appendix 3, 5 of the predictor variables were associated with internet use in the multiple logistic regression analysis, including all 706 respondents. Preferring written information in a language other than English (odds ratio [OR] 0.21, 95% CI 0.12-0.36), lacking confidence in filling out medical forms (OR 0.27, 95% CI 0.11-0.65), being female (OR 0.47, 95% CI 0.26-0.85), and increasing age (OR 0.92, 95% CI 0.90-0.94) were predictive of lower internet use, whereas educational achievement (OR 4.00, 95% CI 1.52-11.07 for college university or higher) predicted greater odds of internet use.

There were 4 independent predictor variables of the use of the internet for health information in the 514 internet users. Females (OR 2.34, 95% CI 1.49-3.71) and people who used the internet several times per day (OR 3.83, 95% CI 2.36-6.30) were more likely to be web-based health information seekers, whereas those lacking confidence in filling out medical forms (OR 0.24, 95% CI 0.07-0.72) and those expressing a preference for written health information in languages other than English (OR 0.53, 95% CI 0.30-0.94) were less likely to be web-based health information seekers (Multimedia Appendix 3).

### Predictors of Digital Device Ownership and Having a Health and Fitness App

A total of 6 predictor variables were associated with ownership of a smartphone or tablet when the whole group was assessed (n=704): educational achievement (college or university or higher: OR 5.44, 95% CI 2.36-12.96) was associated with higher odds of device ownership, while living in Canada for <5 years (OR 0.47, 95% CI 0.27-0.81), preferring written information in languages other than English (OR 0.51, 95% CI 0.31-0.86), having a chronic health condition (OR 0.53, 95% CI 0.31-0.90), having diabetes (OR 0.50, 95% CI 0.28-0.87), and increasing age (OR 0.94, 95% CI 0.92-0.95) were associated with lower odds of device ownership (Multimedia Appendix 3). In the subgroup of smartphone or tablet owners (n=521), only increasing age was associated with lower odds of having downloaded a health and fitness app (OR 0.97, 95% CI 0.95-0.99; Multimedia Appendix 3).

### Predictors of Preferences for Future eHealth Interventions in Internet Users

The multivariable analysis shown in Multimedia Appendix 4 indicated that individuals who preferred written health information in a language other than English were less interested in all modes of eHealth-based support. Those who reported watching YouTube videos were more likely to be interested in a YouTube channel for health issues. Those who are married were more interested in a website with peer support. Interest in app-based interventions was higher in those who own a smartphone or tablet but lower in participants with diabetes. Interest in text message–based interventions was higher in older individuals, those who already send text messages, use the internet several times per day, or are married (Multimedia Appendix 4).

## Discussion

### Principal Findings

Among our sample of primarily Sikh South Asian adults recruited from community and health care settings, we found a high prevalence of internet users and ownership of smart digital devices that allow the use of apps. Health care providers were the most common source of health information, and only less than half of all respondents reported using the internet as a source of health information. Although most smartphone or tablet owners indicated that they used texting, only one-third reported having a health and fitness app on their device. The most commonly used apps were food, diet, or calorie intake trackers. Most internet users indicated that they were likely or very likely to use at least one of the eHealth tools to address a health issue in the next 12 months, and many preferred YouTube videos, a peer-to-peer support website, or smartphone app. Among internet nonusers, lack of technological skills and interest were cited as the most common barriers, and only one-third of these respondents indicated they were likely to get on the web in the future. However, just more than three-fourth of nonusers indicated that they had access to someone who could help them use the internet.

Language preferences, higher educational attainment, and age were common factors driving a digital divide in internet use and digital device ownership in our sample of South Asian adults. Being female, frequent internet use, being confident in filling out medical forms, and preferring written information in English were all positive independent predictors of using the internet for health information purposes in internet users. Age independently influenced whether participants reported having downloaded a health and fitness app. Those who preferred written information in languages other than English showed less interest in all modes of future eHealth support.

### Comparison With Previous Research in the South Asian Community

At the time the survey was conducted in 2014, our study was unique in that it was the first to use a community-based approach, where we mobilized community resources in health, faith gathering, and other settings to explore ownership of digital devices, internet use, and willingness to use eHealth tools specifically among members of the South Asian community in Canada. Furthermore, to our knowledge, it remains to be the only study to explore the predictors of these outcomes in this growing ethnocultural minority group. Data on the use and uptake of technology to address health needs in South Asians in India [41], Sri Lanka [42], and Pakistan [43] have suggested highly variable rates of web-based health information seeking among internet users (ie, 1%-75%). Our findings are comparable with data reported from a 2009-2010 survey of 709 South Asian adults living in the metropolitan Washington DC region, which found that the internet was the leading source of health information (76.9%) [44]. They also found that older participants and those who were US born were more likely to obtain health information from physicians rather than the internet, whereas those who rarely or never speak English at home are more likely to cite friends as a source of health information rather than the internet. We also found that language preference and age were predictors of web-based health information seeking, whereas the duration of time lived in Canada was not a predictor.

### Comparison With Previous Research in the General Population

Previous research exploring variables influencing internet use has identified age [32,45], education [46-49], English language proficiency [47,50], and health literacy [51] as predictors of internet use. Similarly, we found education to be a strong predictor of internet use, whereas preference for South Asian languages (rather than English) predicted lower odds of internet use. We did not find a relationship between internet use and recent immigration [52] or the presence of chronic conditions [53]. An analysis of the 2010 Canadian internet use survey documented that recent immigrants to Canada have lower rates of internet access but that recent immigrants who are on the web have significantly higher levels of web-based activity than Canadian-born residents and earlier immigrants [52]. Although recent studies do not suggest differential internet uptake between males and females [46], in our sample, we found that being female was independently associated with a lower likelihood of internet use. Although gender inequality has existed in South Asian culture [54,55] and may contribute to this difference, males and females in our study gave similar reasons for not being internet users.

Although several theories have been used to explain health information seeking on the internet, the most recent reviews of methods and measures for health information do not provide insight into the factors predicting the uptake of these behaviors [56,57]. Most studies investigating predictors of using the internet for health information purposes identify age, female sex, and educational attainment as independent sociodemographic predictors, whereas other studies have also identified other demographic factors (race, income, and employment), health status, health care access, and digital literacy factors (eg, internet usage intensity) as mediators of web-based health information seeking [57-64]. Although inconsistent, most studies have found that age is a significant predictor of web-based health information–seeking behaviors [58,59,61]. Generally, as age increases, web-based health information seeking decreases; however, the relationship is complex. For example, Veenhof et al [48] documented that Canadians aged 16 to 25 years were significantly less likely to go on the web to search for health-related information than Canadians aged 26 to 65 years. Interestingly, among all respondents in our study, increasing age was a negative predictor of web-based information seeking; however, it was not a significant predictor among internet users. Our finding that female internet users are more likely to be web-based health information seekers is consistent with that reported by others [58-60,62].

Similar to others, we found that smartphone or tablet owners were more likely to be younger, affluent, and highly educated than nonowners [31]. Our finding that 30% of smartphone or tablet owners used health apps is consistent with the range of 19% to 58% reported in studies of racially diverse Americans [36,65,66]. Our finding that younger individuals were more likely to use health apps is consistent with a national survey of 1604 American mobile phone users that found individuals who were younger, had higher income, were more educated, were Latino or Hispanic, and had a BMI in the obese range were more likely to use health apps [66].

Finally, several studies have explored willingness to get on the web and future use of eHealth tools [67-73]. Our finding that 32% of noninternet users thought they would go on the web is higher than the 8% who said they would like to start using the internet or email in a recent Pew Research Centre survey [67]. Encouragingly, 67% of noninternet users reported that they would likely go on the web in the future, indicating that they would be willing to take a workshop or learn from a friend or family member (72%). Other surveys have reported varying levels of interest in specific eHealth interventions, from highs of 83% of women willing to participate in an internet-based postpartum weight loss intervention [68] to lows where only 18% preferred to learn health, wellness, and lifestyle information from a mobile app [70]. Recently, a qualitative study of British South Asians suggested that short text messages to support medication adherence for type 2 diabetes would be acceptable and relevant [74]. Although limited research exists, language preferences and age have been found to predict willingness to use internet or smartphone app–based interventions for health [71,73], consistent with our finding that increasing age is a negative predictor of app use.

### Clinical Implications

First, our results suggest that community-based, culturally tailored strategies would be welcomed and are required to reduce identified digital divides and increase the uptake and use of credible web-based and app-based resources for health purposes among South Asian adults who are current internet users and nonusers. This is particularly timely, given the need to consider and increase remote delivery of health care based on social distancing as a result of the COVID-19 pandemic. Although eHealth and mHealth interventions appear to be more likely to reach certain subgroups of individuals, such as those that are younger, English speaking, and with high educational achievement, particular attention must be paid not to exacerbate health inequities based on these digital divides. Although most internet users were interested in YouTube or web-based peer support interventions, a range of eHealth interventions will be needed to meet the needs of various subgroups within the community. Device and internet training offered in a culturally relevant way for noninternet users who are interested in getting on the web may reduce identified divides, whereas different means, such as using friends or family as intermediaries, will be required to reach noninternet users, particularly those who have no intention to get on the web. Second, as web-based resources are not designed to replace health professional interactions [75], health care professionals and health organizations must play an important role in referring and supporting patients to access credible eHealth resources, including those that are tailored specifically to South Asian health needs.

### Limitations

Our study has several limitations. First, as nonprobability (ie, convenience) sampling was used, selection bias and sampling error make generalizability to the larger South Asian population a concern. We did not translate our survey into other commonly spoken languages (eg, Hindi, Urdu), and our results primarily pertain to the English- and Punjabi-speaking Sikh community. Second, our data were collected in 2014 and are likely not reflective of evolution in the use or ownership patterns. Third, the survey tool itself is not validated; however, most questions were sourced from existing large national surveys or other validated surveys. We recognize that the question relating to language preference for written health information could have been improved by instead asking about the primary language spoken in the home and that the use of a single health literacy screening question, rather than a full health literacy questionnaire, is not optimal. Although smoking is a well-established risk factor for CVD [76], we did not ask about smoking status or use of web-based health information or apps for smoking cessation as part of our survey. This was based on evidence that South Asian Canadians are less likely to be current or former smokers than Canadians of European descent [77] and that smoking is very rare among South Asian Canadian women [78]. Interestingly, a survey conducted around the same time as ours in British Columbia, Canada, also suggested that smoking rates are considerably lower in South Asians than in the general population (never smokers: 87% vs 59%) [79], as does other Canadian research [3]. However, our exclusion of smoking status may be an oversight, as surveys may fail to accurately capture the use of culturally specific smokeless tobacco products [80]. Furthermore, although the survey was translated into Punjabi and formally pretested, community volunteers were trained to administer the survey in Punjabi and 2 volunteers administered just more than 50% of the surveys, there may have been issues with conceptual translation and variability in survey administration. We had some issues with the early version of the survey administered on paper, which resulted in missing data for certain items. Fourth, volunteer and social desirability bias may overinflate our estimates of device ownership, internet use, and willingness to use future eHealth tools. In addition, self-report may have introduced recall bias in outcome and demographic variables. Fifth, the questions relating to likeliness to use eHealth interventions in the future were hypothetical and therefore may overestimate actual willingness had we asked participants to participate in a pilot test of eHealth interventions. Finally, we did not explore differences in survey responses by survey mode or by recruitment location.

### Conclusions

Our survey provides insights into digital divides according to language preferences, education, age, and sex in an ethnocultural minority community in Canada. The high overall rates of internet use for health information, digital device ownership, and interest in eHealth-based interventions in internet users along with high access to individuals who could help them use the internet among nonusers suggest that eHealth supports are feasible among segments of English- or Punjabi-speaking South Asian adults. There is an opportunity for health care providers and health organizations to enhance the use of reliable and culturally relevant eHealth resources to promote health, prevent chronic disease, and support self-management of chronic health conditions for South Asian adults.

## Appendix

Multimedia Appendix 1 e-Patient Project Survey.

Multimedia Appendix 2 Web-based health information–seeking patterns and preferences for future eHealth interventions in internet users.

Multimedia Appendix 3 Predictors of being an internet user, using the web for health information, smartphone or table ownership, and having health and fitness apps.

Multimedia Appendix 4 Determinants associated with being likely or very likely to use different modes of eHealth support in the future in internet users.

## Abbreviations

AUC: area under the curve
CVD: cardiovascular disease
mHealth: mobile health
OR: odds ratio
VIF: variance inflation factor
