# Supplementary material for: Prevalence and Predictors of Health-Related Internet and Digital Device Use in a Sample of South Asian Adults in Edmonton, Alberta, Canada: Results From a 2014 Community-Based Survey
Source: JMIR Public Health Surveill. 2021 Jan 8;7(1):e20671. doi: 10.2196/20671 (PMC7822722; doi:10.2196/20671)
Supplement: Multimedia Appendix 1 [file publichealth_v7i1e20671_app1.docx]

**Appendix 1. e-Patient Project Survey**

**DATA COLLECTOR**

Please select your name

**LOCATION**Please select the location where the survey is being conducted.

**BACKGROUND**

Hello, my name is _________ . I am a volunteer with the University of Alberta. We are doing a short survey about how South Asian adults in Alberta are using the Internet, smartphones, and applications (Apps) in everyday life and for health. The information provided will help to shape future programs that will benefit you, your family, and your community.

Just so you know, everything that you tell me will be kept strictly confidential. The survey will take between 15 and 20 minutes and is voluntary; you can skip any question that you don’t feel comfortable answering. There are no right or wrong answers to the questions, we just want your opinion.” If you participate, your name will be entered into a draw for an iPad Mini.

**SCREENING QUESTIONS**

Would you like to participate?

- Yes
- No

Q. Are you 18 years or older?

- 18 or Older (1)
- Under 18 (2) **** IF PERSON IS NOT 18 OR OLDER STOP THE SURVEY. “THIS SURVEY IS FOR PEOPLE OVER 18. THANK YOU FOR YOUR TIME.”**

Q. Are the ethnic or cultural origins of your family in India, Pakistan, Bangladesh, Nepal or Sri Lanka?

- Yes (1)
- No (2) **** IF PERSON IS NOT SOUTH ASIAN. “THIS SURVEY IS FOR PEOPLE OF SOUTH ASIAN BACKGROUND. THANK YOU FOR YOUR TIME.”**

**DEVICE OWNERSHIP**

Q. Do you have a desktop or laptop computer at home that is connected to the internet?

- Yes (1)
- No (2)
- **(DO NOT READ)** Don't know (4)
- **(DO NOT READ)** No Answer (5)

Q. Do you own a cell phone, or an iPhone or Blackberry or other device that is also a cellphone?

- Yes (1)
- No (2) **(SKIP TO QUESTION 13)****
- **(DO NOT READ)** Don't know (3)
- **(DO NOT READ)** No answer (4)

Q. Some cell phones are called "smartphones" because of certain features they have. Is your cell phone a smart phone such as an iPhone, Android, Blackberry or Windows phone?

- Yes, Smartphone (1)
- No, not a smartphone (2)
- **(DO NOT READ)** Not sure/don't know (3)
- **(DO NOT READ)** No answer (4)

Q. Do you own an iPad or other tablet computer such as an Android Tablet, Microsoft surface or Kindle Fire?

- Yes (1)
- No (2)
- **(DO NOT READ)** Don't know (3)
- **(DO NOT READ)** No answer (4)

**INTERNET USE**

Q. Do you go online, with a computer, cellphone, iPad or other mobile device at least occasionally?

- Yes (1)
- No (2)
- **(DO NOT READ)** Don't know (3)
- **(DO NOT READ)** No Answer (4)

Q. Do you send or receive email, at least occasionally?

- Yes (1)
- No (2)
- **(DO NOT READ)** Don't know (3)
- **(DO NOT READ)** No answer (4)

**NON – INTERNET USERS**

Q. Based on your answers so far it sounds like you are **NOT** really online. What are the reasons you do not go ONLINE?

|  | Yes (1) | No (2) | Don't know (3) | No Answer (4) |
| --- | --- | --- | --- | --- |
| Cost (1) |  |  |  |  |
| Limited access to a computer (2) |  |  |  |  |
| No interest (3) |  |  |  |  |
| Lack of skills (4) |  |  |  |  |
| Privacy reasons (5) |  |  |  |  |
| Fear of technology (6) |  |  |  |  |
| It’s too late to learn (7) |  |  |  |  |
| Because of a disability (8) |  |  |  |  |
| Uncomfortable using a computer (e.g. eye or back strain) (9) |  |  |  |  |
| Some other reason (10) |  |  |  |  |

Q. Are you likely to start going online in the future?

- Yes, within 6 months (1)
- Yes, within 6-12 months (2)
- Yes, in more than a year (3)
- Not likely (4)
- Never (5)

If you are likely to start going online in the future…

Q. How likely are you to use the following strategies to improve your ability to go online with a computer, iPad, smartphone or other digital device?

|  | Not at all (1) | Not likely (2) | Not sure (3) | Likely (4) | Very likely (5) | No Answer (6) |
| --- | --- | --- | --- | --- | --- | --- |
| Attend a hands-on workshop where you could learn more (1) |  |  |  |  |  |  |
| Talk with a friend or family member who can show you more (2) |  |  |  |  |  |  |

Q. If you needed to go online to do something, do you know someone who could help you?

- Yes (1)
- No (2)
- **(DO NOT READ)** Don't know (3)
- **(DO NOT READ)** No answer (4)

**MOBILE PHONE TASKS**

If you own a cellphone or an iPhone or Blackberry or other device….

Q. Do you use your cellphone or tablet to send or receive text messages?

- Yes (1)
- No (2)
- **(DO NOT READ)** Don't know (3)
- **(DO NOT READ)** No answer (4)

Q. In the next 12 months, how likely are you to UPGRADE to a smartphone, such as an

iPhone, or Android?

- Not at all
- Not likely
- Likely
- Very Likely
- Not Sure
- Choose to Not answer

**HEALTH APPLICATIONS USE**

If you own a cellphone, smartphone or tablet computer…

Q. On your SMARTPHONE or TABLET, do you happen to have any health or fitness software Applications (Apps).(e.g., Track your food intake, weight, physical activity, or keep track of your

medications.)

- Yes (1)
- No (2)
- **(DO NOT READ)** Don't know (3)
- **(DO NOT READ)** No answer (4)

If YES is selected…

Q. What TYPE of health and fitness apps are you **currently using**? (Select all that apply)

- Tracking food, diet, or calorie intake
- Monitoring weight
- Physical activity tracking
- Track runs that you take
- Mobile pedometer
- Keep track of medications
- Blood pressure
- Blood sugar or diabetes
- Research or diagnose medical conditions
- Stress management
- Communicate with your doctor or health professional
- Monitor sleep
- Other (please specify)

**SOURCES OF HEALTH INFORMATION**

Q. Where do you get information about health questions that you have? (Select all that apply)

|  | Yes (1) | No (2) | Don't know (3) | No Answer (4) |
| --- | --- | --- | --- | --- |
| Friends (1) |  |  |  |  |
| Family (2) |  |  |  |  |
| Your doctor or other health care provider (3) |  |  |  |  |
| On the internet (i.e., Online) (4) |  |  |  |  |
| TV or Radio (5) |  |  |  |  |
| Books, magazines, or Newspapers (6) |  |  |  |  |
| Others who have the same health condition |  |  |  |  |

**SOUTH ASIAN TAILORED HEALTH INFORMATION**

Q. How important is it for you to find health information tailored to your specific needs as someone of South Asian background?

- Not at all important
- Very unimportant
- Neither important nor Unimportant
- Very important
- Extremely important

Q. In what language would you prefer to receive written health information?

- English (1)
- Punjabi (2)
- Hindi (3)
- Urdu (4)
- Other (5) ____________________
- **(DO NOT READ)** No Answer (6)

**FAMILY MEMBER LOOKED ONLINE ON YOUR BEHALF**

Q. In the past 12 months, has a family member or close friend looked for health information ONLINE on your behalf?

- Yes (1)
- No (2)
- **(DO NOT READ)** Don't know (3)
- **(DO NOT READ)** No answer (4)

**eHealth Literacy Assessment (eHEALS)**

Q. How **important** is it for you to be able to access health information online?

- Not important at all (1)
- Not important (2)
- Unsure (3)
- Important (4)
- Very Important (5)
- **(DO NOT READ)** No answer (5)

Q. How **useful** do you feel going online is in helping you in making decisions about your health?

- Not useful at all (1)
- Not useful (2)
- Unsure (3)
- Useful (4)
- Very useful (5)
- **(DO NOT READ)** No answer (5)

Q. To what extent do you agree or disagree with the following statements?

|  | Strongly Disagree (1) | Disagree (2) | Not Sure (3) | Agree (4) | Strongly Agree (5) | No Answer (6) |
| --- | --- | --- | --- | --- | --- | --- |
| I know **what** health resources are available online. (1) |  |  |  |  |  |  |
| I know **where** to find helpful health resources online. (2) |  |  |  |  |  |  |
| I know **how** to find helpful health resources online.  (3) |  |  |  |  |  |  |
| I know **how to go online** to answer my questions about health. (4) |  |  |  |  |  |  |
| I know how to **use the health information** I find online to help me.(5) |  |  |  |  |  |  |
| I have the skills I need to **evaluate** the health resources I find online. (6) |  |  |  |  |  |  |
| I can tell **high quality** from **low quality** health resources online. (7) |  |  |  |  |  |  |
| I feel **confident** in using online information to make health decisions. (8) |  |  |  |  |  |  |

**HEALTH LITERACY**

Q. How confident are you filling out medical forms by yourself?

- Extremely (1)
- Quite a bit (2)
- Somewhat (3)
- A little bit (4)
- Not at all (5)
- **(DO NOT READ)** No answer (6)

**GENERAL HEALTH QUESTIONS “**Now I would like to ask you a few questions about your general health.”

Q. In general, how would you rate your own health in the past 4 weeks?

- Excellent (1)
- Very Good (2)
- Good (3)
- Fair (4)
- Poor (5)
- **(DO NOT READ)** No answer (6)

Q. Have you ever been told by a doctor, nurse or other health care professional that you have…

|  | Yes (1) | No (2) | Don't Know (3) | No Answer (4) |
| --- | --- | --- | --- | --- |
| Diabetes or sugar disease (1) |  |  |  |  |
| High blood pressure (2) |  |  |  |  |
| Lung conditions (asthma, bronchitis) (3) |  |  |  |  |
| Heart disease (e.g., angina, heart attack, or stroke) (4) |  |  |  |  |
| Cancer (5) |  |  |  |  |
| Arthritis (6) |  |  |  |  |
| Any other chronic health problem that you take medications for everyday (7) |  |  |  |  |
| High Cholesterol (8) |  |  |  |  |

**DIABETES**
Q. How long have you been diagnosed with Diabetes or Sugar Disease?

- Less than 1 year
- 1-5 years
- 6-10 years
- More than 10 years
- Can’t remember
- Choose to Not answer

**DEMOGRAPHICS**

**“**We are now at the last series of questions and the survey is almost done…”

Q. Gender **(DO NOT ASK, JUST RECORD IF MALE OR FEMALE)**

- Male (1)
- Female (2)

Q. What is your age? _____

Q. What is the highest level of education you have completed to date?

- Primary School (grades 1 to 6) (1)
- Secondary School (grades 7 to 12) (2)
- College or University (3)
- Graduate School (4)
- No formal education (5)
- **(DO NOT READ)** No answer (6)

Q. How long have you lived in Canada?

- Canadian born (1)
- 0-5 years (2)
- 6-15 years (4)
- 16-30 years (5)
- Over 30 years (6)
- **(DO NOT READ)** No answer (7)

Q. What is your marital status?

- Single (1)
- Married (2)
- Divorced (3)
- Widowed (4)
- Separated (5)
- Common Law (6)
- **(DO NOT READ)** No Answer (8)

Q. Which South Asian community do you identify with?

- Sikh (1)
- Hindu (2)
- Ismaili (3)
- Muslim (4)
- Other (5)
- **(DO NOT READ)** No Answer (6)

Thank you for completing the ePatient Project Survey.

**INTERNET USE FREQUENCY**

Q. About how often do you go online?

- Several times a day (1)
- About once a day (2)
- 3-5 days a week (3)
- 1-2 days a week (4)
- Every few weeks (5)
- Less often (6)
- **(DO NOT READ)** Don't know (7)
- **(DO NOT READ)** No answer (8)

**INTERNET USE – GENERAL TASKS**

Q. Based on your answers so far it sounds like you **ARE** online. Do you do any of the following things ONLINE, from a computer, smartphone, iPad or other mobile device.

|  | Yes (1) | No (2) | Don't know (3) | No answer (4) |
| --- | --- | --- | --- | --- |
| Read the news or sports (1) |  |  |  |  |
| Use social media sites like Facebook (2) |  |  |  |  |
| Use Twitter (3) |  |  |  |  |
| Watch videos on YouTube (4) |  |  |  |  |
| Listen to music (5) |  |  |  |  |
| Make video calls on Skype for example (6) |  |  |  |  |
| Play online games (7) |  |  |  |  |

**INTERNET USERS – HEALTH INFORMATION ONLINE**

Q. In the past 12 months, have you looked ONLINE for information about the following things either for yourself or someone else:

|  | Yes (1) | No (2) | Don't Know (3) | No Answer (4) |
| --- | --- | --- | --- | --- |
| Symptoms you are experiencing (1) |  |  |  |  |
| Medical treatments or procedures (2) |  |  |  |  |
| Healthy lifestyles such as food choices or exercise, or how to control your weight (3) |  |  |  |  |
| Alternative therapies like herbal medicines, or acupuncture (4) |  |  |  |  |
| A drug or medications (5) |  |  |  |  |
| A specific disease or medical condition (6) |  |  |  |  |

Q. In the past 12 months, has the information you found online:

|  | Yes (1) | No (2) | Don't Know (3) | No Answer (4) |
| --- | --- | --- | --- | --- |
| Affected a decision about how to treat an illness or condition?(1) |  |  |  |  |
| Changed your overall approach to maintaining your health?(2) |  |  |  |  |
| Led you to ask your doctor new questions? (3) |  |  |  |  |
| Led you to go see your doctor? (4) |  |  |  |  |

Q. In the past 12 months, have you done any of the following things ONLINE, either for yourself or someone else?

|  | Yes (1) | No (2) | Don't Know (3) | No Answer (4) |
| --- | --- | --- | --- | --- |
| Read someone else’s experiences about health or medical issues on an online blog, newsgroup or website? (1) |  |  |  |  |
| Watched an online video about health or medical issues? (2) |  |  |  |  |
| Gone online to find others who might have health concerns similar to yours? (3) |  |  |  |  |
| Communicated with your doctor or health care provider online?(4) |  |  |  |  |
| Consulted online rankings or reviews of doctors or other providers (5) |  |  |  |  |
| Posted a health-related question or shared your own personal health experience online (6) |  |  |  |  |
| Joined and online patient support network |  |  |  |  |

**LIKELINESS TO USE TECHNOLOGY FOR CHRONIC DISEASES**

Q. In the next 12 MONTHS, if the following resources were readily available, how likely

would you be to...

|  | Not at all (1) | Not likely (2) | Likely (4) | Very likely (5) | Not sure (3) | No Answer (6) |
| --- | --- | --- | --- | --- | --- | --- |
| Access a **webpage** for South Asians that includes a forum where you could connect with others like you |  |  |  |  |  |  |
| Access a **YouTube** Channel for people with your conditions(s) that has experts talking about best management |  |  |  |  |  |  |
| Use a **smartphone app or wearable device** that can monitor your condition, track your progress on your health goals, and/or provide reminders about when to take your medications |  |  |  |  |  |  |
| Follow a specific **Twitter** account for your condition(s) providing tailored information for South Asians |  |  |  |  |  |  |
| Sign up for personalized **text messages** providing health updates or reminders for your condition(s) |  |  |  |  |  |  |
| Use an online education program |  |  |  |  |  |  |

**LIKELINESS TO USE TECHNOLOGY FOR DIABETES**

Q. In the next 12 MONTHS, if the following resources were readily available, how likely

would you be to...

|  | Not at all (1) | Not likely (2) | Likely (4) | Very likely (5) | Not sure (3) | No Answer (6) |
| --- | --- | --- | --- | --- | --- | --- |
| Access a diabetes **webpage** for South Asians that includes a forum where you could connect with others like you |  |  |  |  |  |  |
| Access a **YouTube** Channel for South Asians with diabetes that has experts talking about how to best manage diabetes |  |  |  |  |  |  |
| Use a **smartphone app or wearable device** that can monitor your condition, track your progress on your health goals, and/or provide reminders about when to take your medications |  |  |  |  |  |  |
| Follow a specific diabetes **Twitter a**ccount providing tailored information for South Asians |  |  |  |  |  |  |
| Sign up for personalized **text messages** providing health updates or reminders about diabetes |  |  |  |  |  |  |
| Use an online education program about diabetes |  |  |  |  |  |  |

**LIKELINESS TO USE TECHNOLOGY FOR HEALTH - LIFESTYLE**

Q. In the next 12 MONTHS, if the following resources were readily available, how likely

would you be to...

|  | Not at all (1) | Not likely (2) | Likely (4) | Very likely (5) | Not sure (3) | No Answer (6) |
| --- | --- | --- | --- | --- | --- | --- |
| Access a **webpage** for South Asians that includes a forum where you could connect with others like you |  |  |  |  |  |  |
| Access a **YouTube** Channel that has local experts talking about health issues |  |  |  |  |  |  |
| Use a **smartphone app or wearable device** |  |  |  |  |  |  |
| Follow a specific **Twitter a**ccount providing health information for South Asians |  |  |  |  |  |  |
| Sign up for personalized **text message** health updates or reminders |  |  |  |  |  |  |
| Use an online education program |  |  |  |  |  |  |
