# Supplementary material for: Prevalence and Predictors of Health-Related Internet and Digital Device Use in a Sample of South Asian Adults in Edmonton, Alberta, Canada: Results From a 2014 Community-Based Survey
Source: JMIR Public Health Surveill. 2021 Jan 8;7(1):e20671. doi: 10.2196/20671 (PMC7822722; doi:10.2196/20671)
Supplement: Multimedia Appendix 2 [file publichealth_v7i1e20671_app2.docx]

**Multimedia Appendix 2.** Web-based health information–seeking patterns and preferences for future eHealth interventions in internet users.

| Characteristic | | Internet users, n (%) | Missing data, n (%) | |
| --- | --- | --- | --- | --- |
| **Frequency of internet use (n=517)** | | | | |
|  | Several times per day | 373 (72.1) | | 10 (1.9) |
|  | Once per day or less | 144 (27.9) | | 10 (1.9) |
| **Do you do any of the following on web?** | | | | |
|  | Watch YouTube videos (n=523) | 425 (80.3) | 4 (0.8) | |
|  | Use social media sites (n=524) | 389 (74.2) | 3 (0.6) | |
|  | Use Twitter (n=523) | 95 (18.2) | 4 (0.8) | |
|  | Listen to music (n=523) | 394 (75.3) | 3 (0.6) | |
|  | Read news or sports (n=522) | 380 (72.8) | 5 (0.9) | |
|  | Make video calls (n=521) | 306 (58.7) | 6 (1.1) | |
|  | Play web-based games (n=523) | 152 (28.4) | 4 (0.8) | |
| **Have you looked on web for information about the following things for yourself or someone else?^a^** | | | | |
|  | Healthy lifestyle (n=524) | 354 (67.6) | 3 (0.6) | |
|  | Specific disease or medical condition (n=460) | 248 (53.9) | 67 (12.7) | |
|  | Medical treatments or procedures (n=520) | 220 (42.3) | 7 (1.3) | |
|  | Symptoms you are experiencing (n=523) | 222 (42.4) | 4 (0.8) | |
|  | Drug or medication (n=519) | 190 (36.6) | 8 (1.5) | |
|  | Alternative therapies (n=521) | 134 (25.7) | 6 (1.1) | |
|  | At least one of the above tasks (n=527) | 455 (86.3) | N/A^b^ | |
| **Have you done any of the following on the web, either for yourself or someone else?** | | | | |
|  | Web-based video about health or medical issues (n=523) | 240 (45.9) | 4 (0.8) | |
|  | Read health or medical blog, newsgroup, or website (n=523) | 222 (42.4) | 4 (0.8) | |
|  | Find others with similar condition (n=518) | 153 (29.5) | 9 (1.7) | |
|  | Communicate with HCP^c^ (n=521) | 67 (12.9) | 6 (1.1) | |
|  | Consulted reviews of doctors or other HCP (n=454) | 36 (7.9) | 73 (13.9) | |
|  | Posted a question on the web (n=260) | 6 (2.3) | 267 (50.7) | |
|  | Web-based peer network (n=240) | 4 (1.7) | 287 (54.5) | |
| **Has the information you found or the information someone else found for you on the web^a^ (n=455;** ≥**1 web-based health information activity)** | | | | |
|  | Changed the way you maintain your health (n=344) | 183 (53.2) | 111 (24.4) | |
|  | Led you to go see your doctor (n=346) | 147 (42.5) | 109 (24.0) | |
|  | Led you to ask your doctor new questions (n=345) | 120 (34.8) | 110 (24.2) | |
|  | Affected a treatment decision (n=343) | 94 (27.4) | 112 (24.6) | |
| **If the following resources were readily available, how likely would you be to (likely or very likely)** | | | | |
|  | At least one (n=527) | 441 (83.7) | N/A | |
|  | Access a YouTube channel for people with your condition that has experts talking about how best to manage it (n=425)^e^ | 330 (77.6) | 102 (19.4%) | |
|  | Access a webpage for South Asians with your health condition that includes a forum to connect with others like you (n=500) | 353 (70.6) | 27 (5.1) | |
|  | Use an app or wearable device to monitor your condition, track your progress on your health goals, and/or provide reminders about when to take your medications (n=493) | 316 (64.1) | 34 (6.5) | |
|  | Sign up for personalized text messages providing health updates or reminders for your condition (n=483) | 282 (58.4) | 44 (8.3) | |
|  | A web-based education program (n=282) ^f^ | 105 (36.7) | 245 (46.5) | |
|  | Follow a specific Twitter account for your condition providing tailored information for South Asians (n=198)^g^ | 47 (23.7) | 329 (62.4) | |

^a^Time frame was the past 12 months.

^b^N/A: not applicable.

^c^HCP: health care provider.

^d^Data are n (%) out of 527 internet users unless otherwise specified. When there were missing data, the n (%) of valid cases was reported.

^e^ YouTube was missed as an option in the paper version of the survey.

^f^The web-based education program was inadvertently in the chronic health condition stream of the computer-assisted personal interview and web-based version of the survey and reflects respondents who did not have a chronic condition.

^g^Twitter was missed as an option in the paper version of the survey and inadvertently in the lifestyle stream of the web-based survey (ie, the responses reflect those with a chronic condition).
