# Supplementary material for: Prevalence and Predictors of Health-Related Internet and Digital Device Use in a Sample of South Asian Adults in Edmonton, Alberta, Canada: Results From a 2014 Community-Based Survey
Source: JMIR Public Health Surveill. 2021 Jan 8;7(1):e20671. doi: 10.2196/20671 (PMC7822722; doi:10.2196/20671)
Supplement: Multimedia Appendix 3 [file publichealth_v7i1e20671_app3.docx]

**Multimedia Appendix 3.** Predictors of being an internet user, using the web for health information, smartphone/table ownership, and having health and fitness apps.

| Characteristic | Internet user (n=706),  OR^a^ (95% CI) | Web-based health information seeker (n=703)^b^,  OR (95% CI) | Web-based health information seeker (n=514 internet users)^c^,  OR (95% CI) | Smartphone or tablet ownership (n=704)^d^,  OR (95% CI) | Health & fitness app downloaded (n=521)^e^,  OR (95% CI) |
| --- | --- | --- | --- | --- | --- |
| **Age (years)** | 0.92 (0.90-0.94) | 0.96 (0.95-0.98) | 0.99 (0.98-1.01) | 0.94 (0.92-0.95) | 0.97 (0.95-0.99) |
| **Sex** | | | | |  |

|  | Male | Referent | Referent | Referent | Referent | Referent |
| --- | --- | --- | --- | --- | --- | --- |
|  | Female | 0.47 (0.26-0.85) | 1.44 (0.98-2.13) | 2.34 (1.49-3.71) | 0.67 (0.41-1.08) | 1.06 (0.70-1.61) |

| **Marital status** |
| --- |

|  | Not married | Referent | Referent | Referent | Referent | Referent |
| --- | --- | --- | --- | --- | --- | --- |
|  | Married | 1.01 (0.43-2.32) | 1.02 (0.62-1.70) | 0.85 (0.48-1.50) | 1.21 (0.62-2.34) | 0.60 (0.36-1.03) |

| **Education** |
| --- |

|  | < High school | Referent | Referent | Referent | Referent | Referent |
| --- | --- | --- | --- | --- | --- | --- |
|  | High school | 1.34 (0.52-3.65) | 2.82 (0.73-18.73) | 2.01 (0.36-16.34) | 2.77 (1.28-6.22) | 0.68 (0.15-4.84) |
|  | ≥ College | 4.00 (1.52-11.07) | 10.78 (2.87-70.84) | 5.10 (0.93-40.62) | 5.44 (2.36-12.96) | 1.76 (0.40-12.32) |

| **Lived in Canada (years)** |
| --- |

|  | >5 | Referent | Referent | Referent | Referent | Referent |
| --- | --- | --- | --- | --- | --- | --- |
|  | 0-5 | 0.60 (0.31-1.18) | 0.71 (0.46-1.01) | 0.88 (0.54-1.43) | 0.47 (0.27-0.81) | 0.72 (0.44-1.15) |

| **Language preference** |
| --- |

|  | English | Referent | Referent | Referent | Referent | Referent |
| --- | --- | --- | --- | --- | --- | --- |
|  | Not English | 0.21 (0.12-0.36) | 0.32 (0.20-0.52) | 0.53 (0.30-0.94) | 0.51 (0.31-0.86) | 0.81 (0.43-1.48) |

| **Community** |
| --- |

|  | Sikh | Referent | Referent | Referent | Referent | Referent |
| --- | --- | --- | --- | --- | --- | --- |
|  | Hindu | 1.56 (0.76-3.31) | 1.32 (0.83-2.13) | 1.37 (0.81-2.35) | 0.78 (0.44-1.40) | 1.05 (0.62-1.76) |
|  | Other | 1.39 (0.46-4.95) | 1.69 (0.88-3.35) | 1.58 (0.78-3.36) | 1.72 (0.66-5.45) | 0.76 (0.39-1.46) |

| **Confidence filling out medical forms** |
| --- |

|  | > Not at all | Referent | Referent | Referent | Referent | Referent |
| --- | --- | --- | --- | --- | --- | --- |
|  | Not at all | 0.27 (0.11-0.65) | 0.22 (0.07-0.55) | 0.24 (0.07-0.72) | 1.05 (0.53-2.13) | 0.63 (0.20-1.69) |

| **Health status** |
| --- |

|  | Excellent | Referent | Referent | Referent | Referent | N/A^f^ |
| --- | --- | --- | --- | --- | --- | --- |
|  | Very good | 1.35 (0.56-3.29) | 1.16 (0.65-2.04) | 1.15 (0.63-2.10) | 1.34 (0.62-2.89) | N/A |
|  | Good | 0.71 (0.33-1.51) | 1.09 (0.64-1.86) | 1.35 (0.75-2.41) | 0.40 (0.36-1.33) | N/A |
|  | Fair | 0.68 (0.27-1.69) | 1.35 (0.69-2.67) | 1.95 (0.89-4.37) | 0.69 (0.32-1.48) | N/A |
|  | Poor | 0.56 (0.14-2.37) | 0.76 (0.24-2.37) | 0.95 (0.27-3.75) | 0.72 (0.22-2.52) | N/A |

| **Chronic condition** |
| --- |

|  | No | Referent | Referent | Referent | Referent | Referent |
| --- | --- | --- | --- | --- | --- | --- |
|  | Yes | 0.76 (0.40-1.46) | 1.05 (0.67-1.65) | 1.16 (0.70-1.91) | 0.53 (0.31-0.90) | 0.78 (0.48-1.26) |

| **Diabetes** |
| --- |

|  | No | Referent | Referent | Referent | Referent | Referent |
| --- | --- | --- | --- | --- | --- | --- |
|  | Yes | 1.04 (0.54-1.99) | 0.90 (0.52-1.55) | 0.89 (0.46-1.71) | 0.50 (0.28-0.87) | 1.24 (0.62-2.58) |

| **Ownership of devices** |
| --- |

|  | No | N/A | N/A | Referent | N/A | N/A |
| --- | --- | --- | --- | --- | --- | --- |
|  | Yes | N/A | N/A | 0.74 (0.35-1.54) | N/A | N/A |

| **Internet use** |
| --- |

|  | ≤1 per day | N/A | N/A | Referent | N/A | N/A |
| --- | --- | --- | --- | --- | --- | --- |
|  | Several per day | N/A | N/A | 3.83 (2.36-6.30) | N/A | N/A |

| **Model fit parameters** |
| --- |

|  | Hosmer-Lemeshow *P* value | .529 | .3642 | .7809 | .6997 | .1197 |
| --- | --- | --- | --- | --- | --- | --- |
|  | Area under the curve | 0.9326 | 0.832 | 0.7707 | 0.8678 | 0.7083 |
|  | Pseudo R^2^ | 0.6405 | 0.4336 | 0.2617 | 0.4439 | 0.1581 |

^a^OR: odds ratio.

^b^Web-based health information seeking (n=3 missing)

^c^Web-based health information seeking in internet users (n=10 missing)

^d^Smartphone or tablet ownership (n=2 missing)

^e^Health and fitness app downloaded in smartphone or tablet owners (n=6 missing).

^f^N/A: not applicable.
