# Supplementary material for: Prevalence and Predictors of Health-Related Internet and Digital Device Use in a Sample of South Asian Adults in Edmonton, Alberta, Canada: Results From a 2014 Community-Based Survey
Source: JMIR Public Health Surveill. 2021 Jan 8;7(1):e20671. doi: 10.2196/20671 (PMC7822722; doi:10.2196/20671)
Supplement: Multimedia Appendix 4 [file publichealth_v7i1e20671_app4.docx]

**Multimedia Appendix 4.** Determinants associated with being likely or very likely to use different modes of eHealth support in the future in internet users.

| Characteristic | | YouTube (n=445)^a^,  OR^b^ (95% CI) | Website (n=494)^c^, OR (95% CI) | App (n=486)^d^, OR (95% CI) | Text messages (n=452)^e^, OR (95% CI) |
| --- | --- | --- | --- | --- | --- |
| Age (years)^f^ | | 1.02 (1.00-1.05) | 1.56 (0.89-2.70) | 0.99 (0.97-1.01) | 1.03 (1.01-1.05) |
| **Sex** | | | | | |
|  | Male | Referent | Referent | Referent | Referent |
|  | Female | 1.22 (0.72-2.07) | 1.46 (0.93-2.32) | 1.12 (0.73-1.73) | 1.37 (0.89-2.12) |
| **Marital status** | | | | | |
|  | Not married | Referent | Referent | Referent | Referent |
|  | Married | 1.23 (0.63-2.36) | 2.14 (1.21-3.77) | 1.53 (0.88-2.67) | 2.33 (1.35-4.05) |
| **Education** | | | | | |
|  | <High school | Referent | Referent | Referent | Referent |
|  | High school | 2.58 (0.41-17.04) | 2.98 (0.57-14.94) | 0.28 (0.05-1.49) | 1.67 (0.19-12.47) |
|  | ≥ College | 4.56 (0.75-28.89) | 4.51 (0.86-22.35) | 0.64 (0.11-3.31) | 1.73 (0.20-12.53) |
| **Lived in Canada (years)** | | | | | |
|  | >5 | N/A^g^ | Referent | Referent | Referent |
|  | 0-5 | N/A | 1.34 (0.82-2.25) | 0.93 (0.58-1.52) | 1.03 (0.64-1.66) |
| **Language preference** | | | | | |
|  | English | Referent | Referent | Referent | Referent |
|  | Not English | 0.44 (0.22-0.93) | 0.35 (0.19-0.64) | 0.42 (0.24-0.73) | 0.43 (0.24-0.78) |
| **Community** | | | | | |
|  | Sikh | N/A | Referent | Referent | Referent |
|  | Hindu | N/A | 0.85 (0.47-1.56) | 0.89 (0.53-1.52) | 0.60 (0.35-1.03) |
|  | Other | N/A | 0.55 (0.28-1.10) | 0.64 (0.33-1.24) | 0.42 (0.22-0.79) |
| **Confidence in filling out medical forms** | | | | | |
|  | > Not at all | Referent | Referent | Referent | Referent |
|  | Not at all | 1.49 (0.46-5.94) | 1.81 (0.57-6.68) | 0.90 (0.33-2.59) | 2.16 (0.72-7.50) |
| **Chronic condition** | | | | | |
|  | No | N/A | Referent | Referent | Referent |
|  | Yes | N/A | 0.78 (0.47-1.30) | 0.62 (0.39-1.00) | 0.84 (0.51-1.38) |
| **Diabetes** | | | | | |
|  | No | Referent | Referent | Referent | Referent |
|  | Yes | 1.14 (0.54-2.32) | 0.51 (0.22-1.10) | 0.47 (0.24-0.90) | 0.94 (0.47-1.85) |
| **Amount of internet use** | | | | | |
|  | ≤1 per day | Referent | Referent | Referent | Referent |
|  | Several per day | 1.73 (0.96-3.10) | 1.60 (0.92-2.75) | 1.51 (0.93-2.44) | 1.74 (1.04-2.90) |
| **Ownership of devices** | | | | | |
|  | No | N/A | Referent | Referent | Referent |
|  | Yes | N/A | 1.05 (1.02-1.07) | 3.29 (1.62-6.87) | 0.92 (0.33-2.44) |
| **YouTube use in general** | | | | | |
|  | No | Referent | N/A | N/A | N/A |
|  | Yes | 2.41 (1.32-4.34) | N/A | N/A | N/A |
| **Social media** | | | | | |
|  | No | N/A | Referent | N/A | N/A |
|  | Yes | N/A | 0.60 (0.24-1.40) | N/A | N/A |
| **Text messages** | | | | | |
|  | No | N/A | N/A | N/A | Referent |
|  | Yes | N/A | N/A | N/A | 2.59 (1.24-5.52) |
| **Model fit parameters** | | | | | |
|  | Hosmer-Lemeshow *P* value | .4338 | .734 | .8421 | .671 |
|  | Area under the curve | 0.6968 | 0.7390 | 0.7037 | 0.7024 |
|  | Pseudo R^2^ | 0.1266 | 0.2020 | 0.1587 | 0.1481 |

^a^YouTube: (n=0 missing). A similar set of predictors was retained in multivariate models regardless of associated *P* values, so the effects could be compared across models. On the basis of the high level of interest in a YouTube-based intervention, this model could only accept 10 to 12 variables, including the intercept, and therefore, this model was condensed.

^b^OR: odds ratio.

^c^Website (n= 6 missing)

^d^App (n=7 missing)

^e^Text messages (n=31 missing)

^f^Owing to the small number of Twitter users (n=50), regression was not performed for this variable. Also, regression on a web-based education program is not presented, as there was a problem in routing for this survey question.

^g^N/A: not applicable.
